# Special Issue on the Promise of Psychedelic Pharmacology in Neuropsychiatric Drug Development

**DOI:** 10.1002/prp2.70309

**Published:** 2026-07-29

**Authors:** Andreas Halman, Ross Corriden

**Affiliations:** ^1^ Peter MacCallum Cancer Centre Melbourne Victoria Australia; ^2^ Sir Peter MacCallum Department of Oncology The University of Melbourne Melbourne Victoria Australia; ^3^ Cancer Therapies, Stem Cell Medicine Murdoch Children's Research Institute Parkville Victoria Australia; ^4^ Pharmacology Research & Perspectives Editorial Board New Castle New South Wales Australia

The scientific study of psychedelics has followed an uneven path over time. After promising psychiatric research on LSD in the 1950s and early 1960s, the field started to decline and came to a near halt by the mid‐1970s, due to increasing legal controls, Schedule I classification and broader changes in pharmaceutical research regulation [1]. Now, half a century later, psychedelic research has regained notable traction as the clinical need for new treatments for intractable depression and post‐traumatic stress disorder has increased significantly. Over the past decade, research studies and clinical trials have increased substantially (Figure 1), reflecting a broader shift in the scientific and clinical approach to these compounds. Alongside this increase in research activity, some jurisdictions, including Australia, New Zealand and some states in the United States, have regulated the use of psychedelics for medicinal use. Further growth may follow recent policy developments in the United States aimed at accelerating research, development and access to psychedelic treatments for serious mental illnesses [2]. The field is therefore moving from rediscovery to translation, where each compound must be evaluated in relation to the appropriate clinical phenotype, with a defined dosing strategy, delivery model and robust evidence. The four papers in this special issue, *Promise of Psychedelic Pharmacology in Neuropsychiatric Drug Development*, examine different points along that pathway: from preclinical evidence on potential new therapeutic avenues to the clinical implementation itself. It shows significant development in the understanding of where the clinical benefit might lie, with increasing knowledge around safety profiles.

**FIGURE 1:**
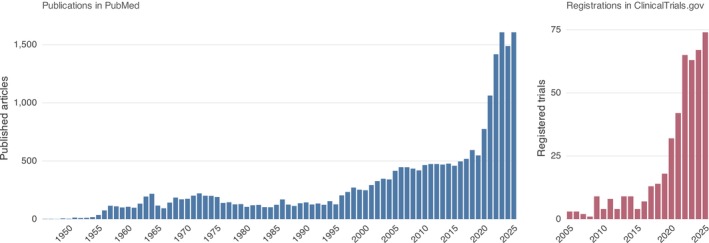
Classic psychedelics, ibogaine and MDMA research activity. Bars show the annual number of PubMed‐indexed publications identified using search terms for classic psychedelics, ibogaine and MDMA, alongside the annual number of ClinicalTrials.gov registrations for trials involving these interventions identified using a similar search.

The renewed interest in psychedelic pharmacology has been shaped largely by the clinical need in psychiatry, but its scientific significance may be broader. This is illustrated by the preclinical work of Balabandian and colleagues, who investigated psilocin in experimental seizure models. Their mouse study suggests that psilocin can attenuate seizure activity in pentylenetetrazole‐ and maximal electroshock‐induced models, with anticonvulsant effects that may be mediated through 5‐HT_1A_ and CB1 receptors, opioidergic and nitrergic systems and the kynurenine pathway [3]. This finding is important as it moves psychedelic pharmacology beyond its current main focus on mood, trauma and substance‐use disorders. It also serves as a reminder that psychedelics, such as psilocin, are not only agents that produce altered states of consciousness, but pharmacologically active compounds whose effects may extend beyond the well‐known 5‐HT_2A_ mediated psychoactivity. Although the work remains preclinical, it opens a new experimental path for further investigation, with potential implications for future clinical research in neurology.

Even so, the field remains largely anchored in psychiatry. Kazdan and colleagues examine comorbid major depressive disorder and chronic pain, highlighting that these conditions should also be considered together rather than treated as isolated disorders [4]. Although research on psychedelics for pain has gained attention, their role in this specific comorbidity remains largely unexplored. Based on the preliminary evidence and shared neurobiological and psychological therapeutic targets in depression and pain, the authors provide a rationale for future studies of psychedelic‐assisted approaches in comorbid major depressive disorder and chronic pain.

While numerous clinical trials are now investigating different psychedelics across a range of conditions, their findings raise an important interpretive challenge. As E. Schenberg discusses, psychedelic trials can be difficult to evaluate because masking is often compromised, expectancy effects may be substantial and the intervention being tested may combine pharmacological effects with psychological and contextual support [5]. This matters because psychedelics as “stand‐alone” medicines and psychedelic‐assisted therapies make different causal claims. Evidence from a combined intervention should not be interpreted as evidence for the pharmacological component in isolation. Therefore, the extent to which efficacy in randomized controlled trials can be trusted to predict effectiveness in real‐world populations depends on the type of psychedelic treatment being assessed. The evidence required should be aligned with the intervention being evaluated.

This distinction becomes even more important as psychedelic‐assisted therapy moves from clinical trials into practice. In line with Schenberg's point, if the evidence comes from a combined intervention, then the treatment delivered in practice needs to reflect that intervention. Trials usually include rigorous screening, safety monitoring, structured preparation, therapeutic support, supportive environments and integration sessions. They also commonly rely on strict inclusion and exclusion criteria and comprehensive assessment before enrolment. These are in place to support participant safety and may also contribute to therapeutic outcomes, but they create challenges for wider implementation. Kim and colleagues address this timely challenge directly, emphasizing that psychedelic‐assisted therapy is more than simply a compound administered in a clinic [6]. Screening, preparation, therapeutic environment, monitoring, psychological support and integration are important components of the intervention. If these elements are removed to reduce cost or improve scalability, the treatment delivered in practice may be less effective and introduce additional risk. The trade‐off between safety, fidelity and access must therefore be carefully weighed. Safe implementation will require regulation that preserves the essential features of psychedelic‐assisted therapy without making access disproportionately difficult.

In summary, the articles in this special issue show that psychedelic pharmacology is entering an important phase, in which research findings are being translated into clinical practice and access to these treatments is expanding. In light of this, and as highlighted throughout this issue, several questions need to be addressed to better understand treatment effectiveness and to ensure that psychedelic therapies are delivered to the right people, at the right dose, for the right conditions and in appropriate clinical settings.

## Author Contributions

**Ross Corriden:** conceptualization, writing – original draft. **Andreas Halman:** conceptualization, writing – original draft, visualization, project administration.

## Conflicts of Interest

The authors declare no conflicts of interest.

## Data Availability

Data sharing not applicable to this article as no datasets were generated or analyzed during the current study.
